# Mass Spectrometry Imaging of Coniine and Other Hemlock Alkaloids after On-Tissue Derivatization Reveals Distinct Alkaloid Distributions in the Plant

**DOI:** 10.1021/acs.jnatprod.4c00445

**Published:** 2024-06-21

**Authors:** Diana A. Barrera-Adame, Sabine Schuster, Timo H. J. Niedermeyer

**Affiliations:** † Department of Pharmaceutical Biology/Pharmacognosy, Institute of Pharmacy, Martin Luther University Halle-Wittenberg, 06120 Halle (Saale), Germany; ‡ Department of Pharmaceutical Biology, Institute of Pharmacy, Freie Universität Berlin, 14195 Berlin, Germany

## Abstract

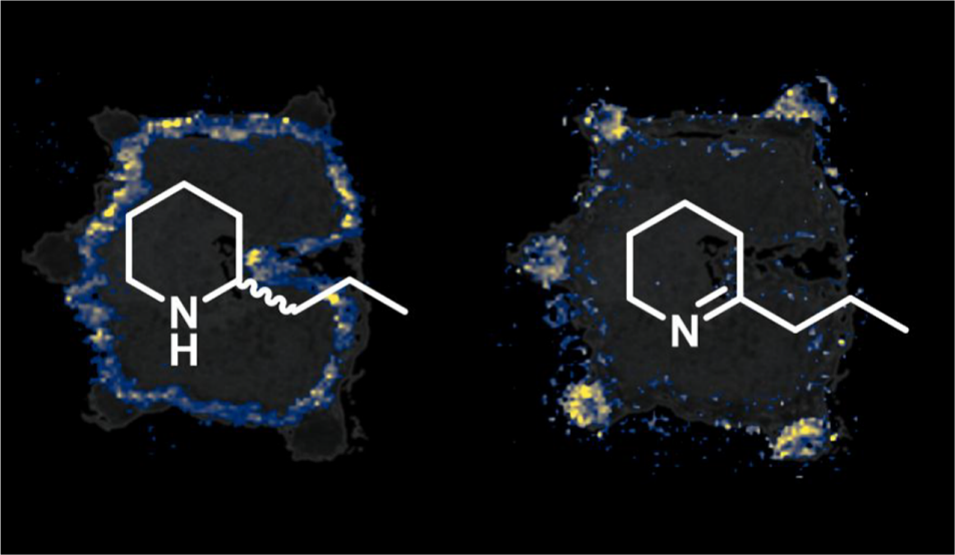

Specialized metabolites play important roles in plants and can, for example, protect plants from predators or pathogens. Alkaloids, due to their pronounced biological activity on higher animals, are one of the most intriguing groups of specialized metabolites, and many of them are known as plant defense compounds. Poison hemlock, *Conium maculatum*, is well-known for its high content of piperidine alkaloids, of which coniine is the most famous. The distribution, localization, and diversity of these compounds in *C. maculatum* tissues have not yet been studied in detail. The hemlock alkaloids are low molecular weight compounds with relatively high volatility. They are thus difficult to analyze on-tissue by MALDI mass spectrometry imaging due to delocalization, which occurs even when using an atmospheric pressure ion source. In this manuscript, we describe an on-tissue derivatization method that allows the subsequent determination of the spatial distribution of hemlock alkaloids in different plant tissues by mass spectrometry imaging. Coniferyl aldehyde was found to be a suitable reagent for derivatization of the secondary amine alkaloids. The imaging analysis revealed that even chemically closely related hemlock alkaloids are discretely distributed in different plant tissues. Additionally, we detected a yet undescribed hemlock alkaloid in *Conium maculatum* seeds.

The poison hemlock, *Conium maculatum*, is a plant native to Europe, northern Africa, and western Asia.^1^ It is one of the most poisonous plants of the northern hemisphere^2^ and has also spread over America and Oceania after it was introduced in these continents as an ornamental plant.^3^ Ingestion of the plant by mammals affects their central nervous system, causing ataxia, tremor, and convulsions.^1,2^ These effects have given the plant its genus name *Conium* (Greek, koneios, spin or whirl), while the species name, *maculatum*, refers to the reddish spots throughout the stem and leaf stalk (Latin, spotted).^1^*C. maculatum* is a biennial plant. In the first year, the plant is in its rosette phase, not producing flowers or seeds, which are raised in the second year.^4^

The plant contains piperidine alkaloids, mainly coniine (**1**) and γ-coniceine (**2**), but also, for example, *N*-methylconiine, conhydrine, pseudoconhydrine, and conhydrinone (**3**).^2,3^**1** has not only been described as a constituent of *Conium maculatum* (Apiaceae) but also from taxonomically unrelated plants such as *Aloe* (Xanthorrheaceae) and *Sarracenia* (Sarraceniaceae) species.^5^ It is famous in history and science: It was used to execute the Greek philosopher Socrates in 399 BCE, and it was the first alkaloid of which the structure has been fully established and which was subsequently synthesized in 1886.^1^

The role of hemlock alkaloids for the plant has not yet been determined. Some authors suggested that these alkaloids could be insect paralyzing agents.^6^ However, others postulated that they serve to attract insects.^5^ Interestingly, the combination of these two effects might serve *Sarracenia purpurea*, an insectivorous plant that contains **1** and has the ability to attract and capture insects.^1,7^**1** is known to act on nicotinic acetylcholine receptors and has pronounced pharmacological effects. It for instance has analgesic effects and can potentiate the analgesic or antinociceptive activity of other compounds.^8^ Accordingly, **1** and its derivatives have received interest concerning a potential use in pharmacy.^1^

The concentrations of **1** and related alkaloids in the plants are highly variable, depending on parameters such as the age of the plant, wet or dry weather, circadian cycle, and/or season.^3^ Studies also revealed that when the concentration of **1** reaches its maximum, **2** is at a minimum and vice versa, due to the fast NADPH+H^+^/NADP^+^-dependent catalytic interconversion between **1** and **2**.^3^ The distribution of the hemlock alkaloids in *C. maculatum* tissues has to date only been studied using color reactions directly on-tissue^9^ and with desorption electrospray ionization mass spectrometry (DESI-MS).^2^ Color reactions have the disadvantage that they do not reveal which specific alkaloid is present at the site where the color develops. Nevertheless, Fairbairn et al. described the presence of a layer in the endocarp of the fruits they named “coniine layer”, where they suggested coniine is stored.^10^ Talaty et al. analyzed various parts of the plant with DESI-MS and revealed the presence and concentration of alkaloids in parts of the plant but without providing information about the specific location of each compound in the tissues.^2^

Direct analysis of compounds on-tissue by mass spectrometry imaging (MSI) has been gaining attention in plant natural product research.^11,12^ However, MALDI-MSI has limited applications for low molecular weight compounds, as conventional matrices have a high background interference in the low-mass range, and thus their signals often overlap with the analyte signals.^13^ The hemlock alkaloids have a comparably high vapor pressure and are thus rather volatile,^2,14^ impeding their direct analysis by MALDI-MSI.

Chemical derivatization methods are routinely used in GC and HPLC coupled to mass spectrometry.^15^ They are still less common in MSI, although chemical derivatization directly on-tissue has many potential applications. Derivatization in MSI can, for example, be used to change physicochemical characteristics of target molecules, such as stability or poor ionizability that result in low signal intensity, or volatility that can result in delocalization.^16^

Amines are functional groups that can conveniently be used for structural modifications. This is based on the high basicity and nucleophilicity of amine groups at higher pH. While aromatic and tertiary amines are less reactive, primary or secondary amines are often suitable for derivatization.^17^ One example of a compound reacting with amines is cinnamaldehyde, which has been used as a derivatization agent on plant tissues to determine the distribution of primary amines.^18,19^ The mechanism of this derivatization is based on the reaction of aldehyde groups with primary amines to form Schiff bases. Another derivatization agent is *p*-*N*,*N*,*N*-trimethylammonioanilyl *N*′-hydroxysuccinimidyl carbamate iodide (TAHS). TAHS has to date only been used on animal tissues to derivatize primary amines in free amino acids,^20−23^ but its reaction with secondary amines such as proline and catecholamines has also been reported.^22−25^ One of the advantages of TAHS derivatization is that it provides a characteristic cleavage signal in tandem mass spectrometry experiments (fragment ion at *m*/*z* 177).^24^ MS/MS analysis has several benefits, including the identification of undescribed compounds by fragmentation pattern comparison between the potentially unknown and standard compounds.^26^ Other reagents that have been used for the derivatization of amines in the past have not been considered further in this study because they were described to react mainly with primary amines, have the potential to form multiple products, or were not readily available with our budget.^15,27,28^

To gain knowledge about the localization and distribution of specific conium alkaloids in the plant, we used MALDI-MSI to analyze their spatial distribution. We demonstrate that chemical on-tissue derivatization of plant alkaloids with coniferyl aldehyde (CA) is suitable for subsequent MALDI-MSI analysis, and that the hemlock alkaloids are discretely distributed in different plant tissues.

## Results and Discussion

### Alkaloid Distribution in *C. maculatum* Fruit Tissue

As previous studies reported that the *Conium maculatum* fruits have the highest alkaloid content within the plant, represented mainly by **2**,^2,29^ we decided to visualize the conium alkaloids **1** to **8** in fruits first.
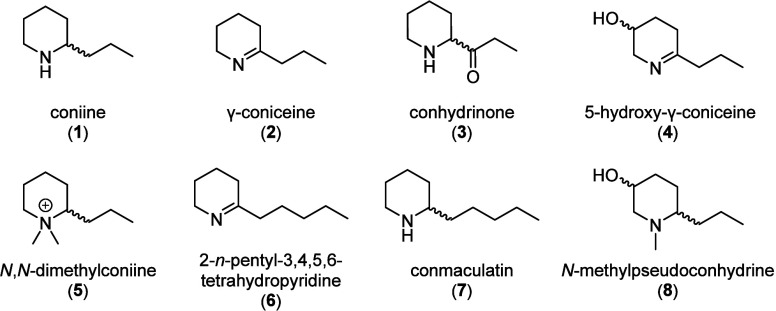


**Figure 1:**
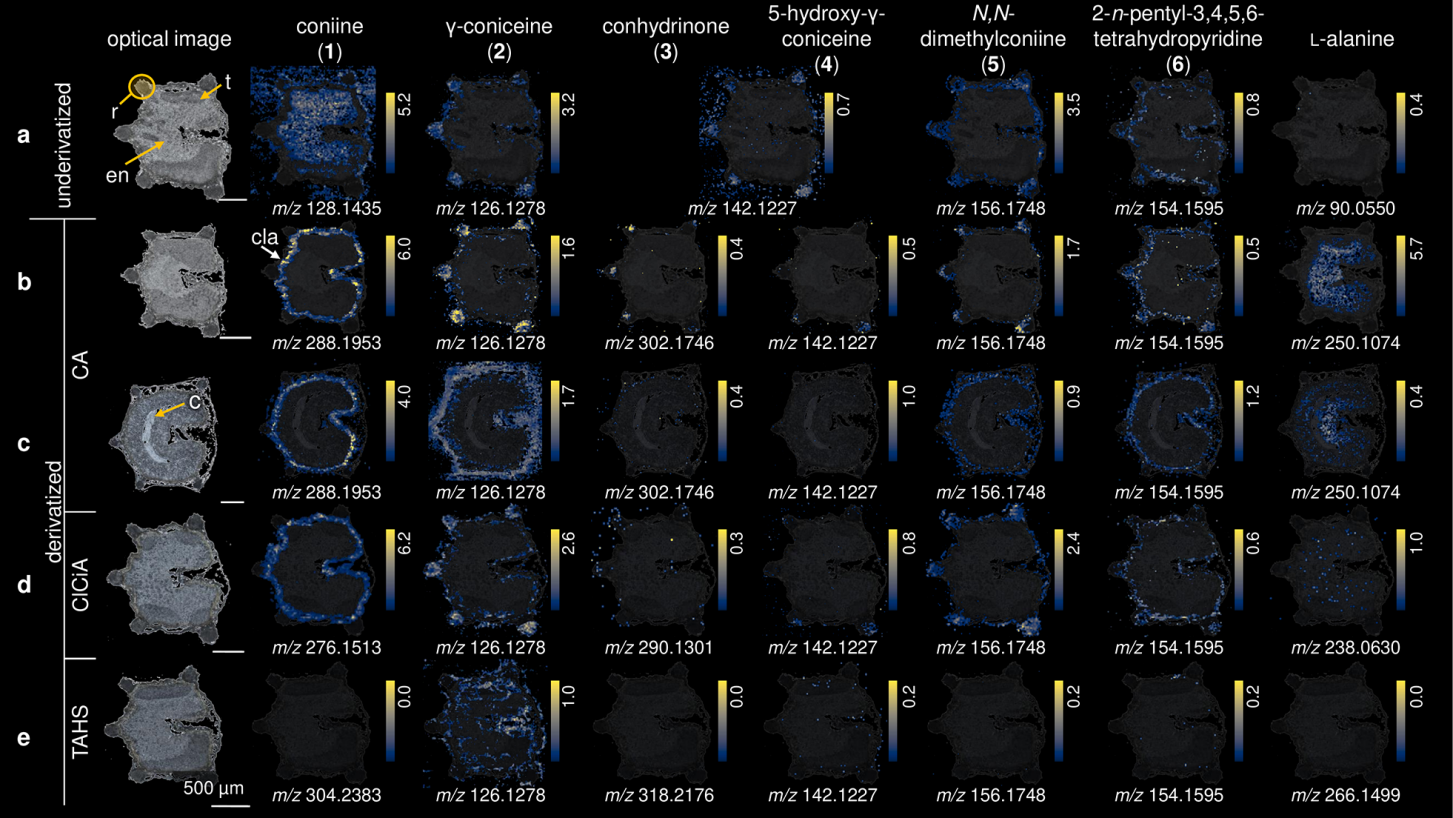
MSI images of *C. maculatum* fruits using different derivatization agents. (a) Underivatized, (b) derivatized with CA, (c) germinated seed derivatized with CA, (d) derivatized with ClCiA, and (e) derivatized with TAHS. Rib (r), testa (t), endosperm (en), “coniine layer” (cla), cotyledon (c). Ion images of individual *m*/*z* values were generated on the same colorbar scale (yellow for the maximum percentage of ions and black for 0 ions detected) for visual comparison in terms of relative percentage of ion abundance.

However, in our first experiments, we found that **1** (detected at *m*/*z* 128.1435, Δ 0.8 ppm) is not amenable for direct analysis using atmospheric pressure MALDI-MSI due to delocalization of **1** in the sample (see Figure 1a, showing the presence of **1** even on the sample stage, outside of the tissue). This delocalization most likely is due to the volatility of **1**, as the sample is in close proximity to the 450 °C hot inlet capillary of the mass spectrometer. The delocalization effect was also observed for conhydrinone (**3**) or 5-hydroxy-γ-coniceine (**4**), detected at *m*/*z* 142.1227 (Δ 0.7 ppm). The latter two alkaloids have the same molecular formula, making it impossible to distinguish them without using tandem mass spectrometry. Surprisingly, γ-coniceine (**2**), detected at *m*/*z* 126.1278 (Δ 0.8 ppm), was found to be discretely localized in the fruit tissues, even though **1** and **2** possess similar structures and boiling points (166 vs 171 °C).

Due to the close chemical similarity of *C. maculatum* alkaloids with *Punica granatum* alkaloids, it was originally suggested that **1** likewise is derived from the amino acid lysine.^30^ However, subsequent studies showed that **1** is biosynthesized via a polyketide synthase, catalyzing the synthesis of the carbon backbone 5-keto-octanal from one butyryl-CoA unit and two malonyl-CoA units.^31^ Transamination using l-alanine and ring closure results in the final product **1**.^1,32^ The fruit has been shown to be a very active organ in the synthesis of alkaloids.^33^ For its biosynthetic relevance, we were interested in also analyzing the spatial distribution of l-alanine (*m*/*z* 90.0550, theoretical mass). However, l-alanine could not be detected in the tissue without derivatization (Figure 1a).

These rather unsatisfying preliminary results—delocalization of the alkaloids, not being able to discriminate between isobaric alkaloids, l-alanine not detectable—prompted us to develop a method for the on-tissue derivatization of the hemlock alkaloids to modify the physicochemical characteristics of these small compounds and increase MALDI-MS signal intensity in positive mode by installing a permanent positive charge. For this aim, the suitability of different derivatization reagents was studied. First, test tube reactions were carried out with **1** and three derivatization agents for amines, CA, 4-chlorocinnamaldehyde (ClCiA), and TAHS. Both CA and ClCiA can potentially be used to derivatize **1** and related secondary alkaloids by formation of iminium ions between the aldehyde group of CA/ClCiA and the amino group in the alkaloids (Scheme S1a/b). Derivatization with CA was used on maize tissues previously, to analyze primary amines (amino acids),^18,19^ but to the best of our knowledge, it has not yet been used to derivatize secondary amine alkaloids on-tissue. ClCiA was selected because of the chlorine atom present in its structure, which, due to its characteristic isotope pattern, would facilitate the identification of the reaction products by MS. ClCiA has not previously been used as derivatization agent for MSI analyses. TAHS (Scheme S1c) possesses a trimethylanilynium moiety that due to its permanent positive charge should improve the signal intensity; this moiety also produces a characteristic fragment ion at *m*/*z* 177.1021 (fragmentation in the ureido group, Figure S3c).^22^ TAHS has not previously been used for on-tissue derivatization of plant tissues. The reaction mixtures of CA, ClCiA, and TAHS with **1** were analyzed by LC-MS (Figures S1 to S3) and MALDI spot analysis for TAHS (Figure S4), showing the effectiveness of the reactions in obtaining the corresponding derivatization products. Subsequently, the reactions were tested directly on the plant tissue.

First, we compared the underivatized tissue (Figure 1a) with the CA-derivatized tissue (Figure 1b). The detection of **1** and **3** was significantly improved after the derivatization. This was most notable for **1** (detected at *m*/*z* 288.1953, [M]^+^, Δ 1.7 ppm). Visualization of the “coniine layer” described by Fairbairn et al. and Corsi et al. confirmed that in this layer indeed coniine is present.^9,10^ With the selected concentration of CA, **1** was not quantitatively derivatized, we could still detect small residual amounts of unreacted **1** on the tissue (Figure S5). However, as increased CA concentration led to reduced sensitivity due to ion suppression, we did not further increase its concentration.

Although **3** was detected with lower ion intensity than **1**, it could be localized to be present in the ribs (Figure 1b). Next to the derivatization product of **3** (*m*/*z* 302.1746, [M]^+^, Δ 1.6 ppm), we could also detect signals (*m*/*z* 142.1227) that correspond either to its underivatized form or to compound **4** ([M + H]^+^). Considering that the formation of the iminium ion product is reversible, it is not possible to discriminate between these species. For that reason, the location of **4** in the fruit is ambiguous, since the signals obtained for derivatized **3** and underivatized **4** (Figure 1b) were both located in the ribs.

Interestingly, the comparison between derivatized and underivatized tissues showed the presence of some other alkaloids. An *m*/*z* signal not assignable to known hemlock alkaloids in *C. maculatum* was detected at *m*/*z* 156.1748 (Figure 1a). The calculated formula, C_10_H_22_N ([M + H]^+^, Δ 0.1 ppm), agrees with *N*,*N*-dimethylconiine or conmaculatin. However, as we could detect derivatized conmaculatin (*m*/*z* 316.2270, [M]^+^) in other parts of the tissue (Figure S6), it can be assumed that the signal *m*/*z* 156.1748 corresponds to the cationic compound *N*,*N*-dimethylconiine (**5**). This alkaloid has been isolated previously from the plant *Aloe sabaea*.^34^**5** was localized in the ribs (Figure 1b). In this part of the tissue and in the “coniine layer”, another analyte was detected at *m*/*z* 154.1595, with the calculated formula C_10_H_20_N ([M + H]^+^, Δ 3.2 ppm). This agrees with 2-*n*-pentyl-3,4,5,6-tetrahydropyridine (**6**), an alkaloid that was only tentatively suggested as a constituent of *C. maculatum* after analysis by electron impact ionization mass spectrometry (EI-MS).^35^ The reduced derivative, conmaculatin (**7**), was previously identified in the roots of *C. maculatum*(36) and detected in our experiments after derivatization (Figure S6). As **1** is derived by reduction of **2**, Hotti et al. hypothesized that **7** is the reduction product of its respective unsaturated biosynthetic precursor, **6**.^31^ Although this alkaloid has a longer aliphatic chain, its biosynthetic route has been proposed as a product of polyketide synthase CPKS5 by condensation of a hexanoyl-CoA unit with two malonyl-CoA units, the same enzyme that catalyzes the biosynthesis of the other hemlock alkaloids.^31^ The compound was located in the endocarp.

In the ribs, and in addition to a certain extent also in the testa, the underivatized alkaloids **2**, **5**, and **6** were detected, which are not reacting with CA. Their distribution was maintained in both the derivatized and underivatized tissues (Figure 1a,b).

Finally, after CA derivatization, l**-**alanine (*m*/*z* 250.1074, [M + H]^+^, Δ 0 ppm, Figure 1b), could be detected in the endosperm, a tissue of the fruit that plays an important role in supplying nutrients, protection, and growth control of the embryo.^37^ This might indicate that l**-**alanine is stored in the endosperm, for future use in hemlock alkaloid biosynthesis or other use by the plant.

After showing the suitability of CA derivatization for the localization of the hemlock alkaloids in ungerminated seed in fruits, we decided to repeat the CA derivatization experiment using a fruit with germinated seed (Figure 1c). In this fruit, it was possible to observe the cotyledons before they grew out from the testa. Curiously, no alkaloids were detected in the cotyledons, suggesting that the germinating plant is protected by the alkaloids that are present in the testa. Interestingly, significant alkaloid distribution differences could be observed in fruits with germinating versus nongerminated seed (Figure 1b,c): The signal intensities of **1** and **3**/**4** decreased noticeably, while production of **2** was massively increased in the testa, probably due to oxidation of **1** to **2**. In addition, **2**, **5**, and **6** showed relocalization from the rib to the testa during germination, possibly to protect the new plant from attack by biotic factors susceptible to its toxicity, which is enhanced by the unsaturation in the piperidine ring.^38,39^ Additionally, l**-**alanine seems to be consumed during the germination process, either to support growth of the cotyledon or to increase the production of **2**.

On ClCiA-derivatized tissues (Figure 1d), we observed that the distributions of **1** at *m*/*z* 276.1513 ([M]^+^, Δ 0.4 ppm) and l**-**alanine at *m*/*z* 238.0630 ([M + H]^+^, Δ 0.4 ppm) were concordant with CA derivatization. Indeed, these results verified the reproducibility of the reactions and the methods. Additionally, for the underivatizable compounds **2**, **5**, and **6**, we observed the same localization as in underivatized and CA-derivatized fruits. However, after ClCiA derivatization, the signal intensity was about 175-fold lower for l**-**alanine and 14-fold lower for **3** (*m*/*z* 290.1301, [M]^+^, Δ 1.7 ppm; average peak intensity in image after CA derivatization 140 and 28, after ClCiA derivatization 0.8 and 2.0 for l**-**alanine and **3**, respectively).

Unfortunately, TAHS derivatization was found not to be suitable on-tissue in our hands: Before carrying out the direct reaction on-tissue, tube reactions were performed in order to standardize the method and to monitor formation of the coniine–TAHS product (*m*/*z* 304.2386, [M]^+^). The reactions were carried out with and without a non-nucleophilic base and were analyzed by MALDI-MS. The results showed that the reaction between **1** and TAHS only occurs in the presence of the base (Figure S4a/b). For this reason, the experiment on-slide was carried out under alkaline conditions with the parameters described by Toue et al.^22^ However, while the reaction on-slide showed the formation of the coniine–TAHS product, we could also detect the hydrolysis product of TAHS at *m*/*z* 151.1233 (Figure S4c). This may indicate that the reaction on-tissue could be impeded by the degradation of TAHS before derivatization occurs. Due to the fact that the best results were obtained for CA, on-tissue derivatization using ClCiA and TAHS were not further optimized.

### Detection of a Potentially Novel Hemlock Alkaloid in *C. maculatum* Fruit

**Figure 2:**
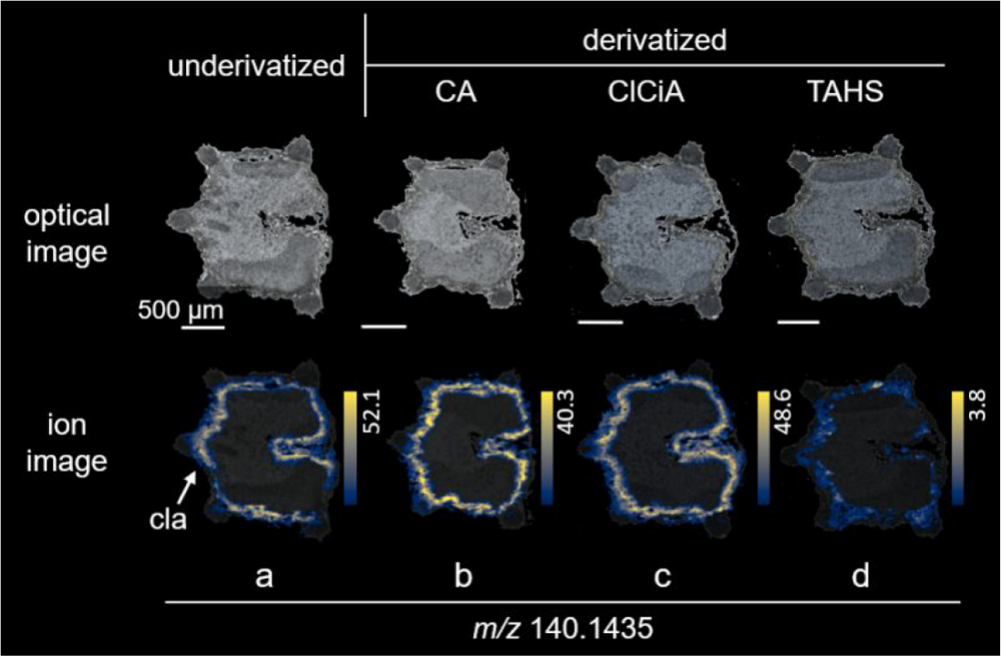
MSI visualization of the compound detected at *m*/*z* 140.1435 in *C. maculatum* fruit tissues. (a) Underivatized, (b) CA, (c) ClCiA, and (d) TAHS derivatization. “Coniine layer” (cla). Ion images of individual *m*/*z* values were generated on the same colorbar scale (yellow for the maximum percentage of ions and black for 0 ions detected) for visual comparison in terms of relative percentage of ion abundance.

While analyzing the MALDI-MSI data of *C. maculatum* fruits, we observed an intense signal at *m*/*z* 140.1435 in the “coniine layer” (Figure 2a), suggesting the presence of a compound with the sum formula C_9_H_18_N (Δ 0.7 ppm). Direct infusion mass spectrometry analysis of a fruit extract and selection of the precursor ion of this compound for fragmentation revealed that this compound also is a piperidine alkaloid similar to coniine and γ-coniceine (characteristic fragment, for example, at *m*/*z* 55.055,^34^Figure S8). A search for this formula in the Dictionary of Natural Products (version 32.2) revealed 2-butyl-1-azacyclohexene, which is a piperidine alkaloid that has been described from the mushroom *Tylopilus* sp. (Boletaceae).^40^ However, its biosynthetic pathway would not involve acetate units only, making its synthesis in *C. maculatum* unlikely.^1^ The signal was observed in derivatized and underivatized tissues (Figure 2), while the masses of the corresponding derivatization products could not be detected. This indicates that the compound does not contain either a primary or a secondary amine.

As the calculated formula does not agree with any of the known conium alkaloids, we hypothesized that this compound might be an artifact formed by dehydration of *N*-methylconhydrine or *N*-methylpseudoconhydrine (**8**). However, this dehydration has not been described previously in analytical studies of 5- and 8-hydroxypiperidine alkaloids,^41−43^ and a detailed analysis of the compound’s MS/MS spectrum indeed indicated neither to be the case: Fragments observed at *m*/*z* 96.0810 and 111.1043, corresponding to the loss of C_3_H_7_ and C_2_H_5_, common in aliphatic chains, indicated that the alkaloid has a saturated side chain and that an unsaturation is located within the heterocycle, ruling out *N*-methylconhydrine as a precursor. A characteristic fragment for **8** at *m*/*z* 114 was absent from our data,^42^ ruling out **8** as a precursor. In addition, closer analysis of the MS imaging data revealed that a compound with a formula corresponding to *N*-methylconhydrine or **8** was located in the ribs, while the compound detected at *m*/*z* 140.1435 was found exclusively in the coniine layer (Figure S7). Roberts et al. described an *S*-adenosylmethionine methyltransferase that is capable of *N*-methylating the alkaloids coniine, conhydrine, and pseudoconhydrine.^33^ Thus, we next hypothesized that *N*-methylconiine might have been oxidized to *N*-methyl-γ-coniceine, which would have the required formula. Detected fragment ions at *m*/*z* 68.0501 and 70.0657, which should be present in the case of *N*-methylated γ-coniceine,^34,44^ would support this hypothesis (Figure S8). However, although the presence of a positively charged nitrogen would explain the observed high ion intensity, this iminium ion would likely be rather unstable and rearrange to the respective enamine. As evaluation of the MS/MS data did not allow us to unequivocally determine the localization of the double bond, the structure of this compound remains elusive.

### CA Derivatization of Alkaloids in Different Tissues of *C. maculatum*

**Figure 3:**
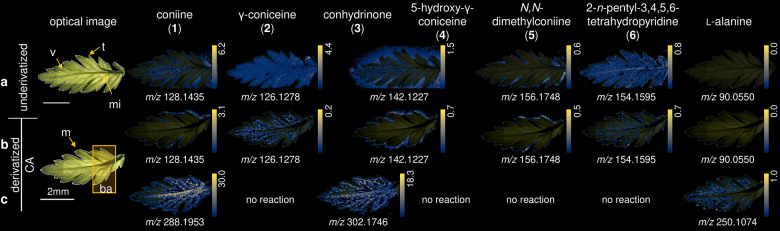
Mass spectrometry images of selected hemlock alkaloids on leaves. (a) Underivatized, (b) CA derivatization with *m*/*z* of underivatized compounds, (c) CA derivatization with *m*/*z* of derivatized compounds. Midrib (mi), veins (v), tips (t), margin (m), and base (ba). Ion images of individual *m*/*z* values were generated on the same colorbar scale (yellow for the maximum percentage of ions and black for 0 ions detected) for visual comparison in terms of relative percentage of ion abundance.

After establishing the derivatization and MSI analysis with *C. maculatum* fruits, we also analyzed the distribution of the conium alkaloids on leaves, a part of the plant that has caused accidental intoxication due to its similarity with parsley.^3^ As can be seen in Figure 3, the derivatization also improves the detection of *C. maculatum* alkaloids on the leaf surface. (Notice the delocalization/poor detectability of **1**, **3**, and l-alanine on the untreated leaf.)

Previous studies showed that the principal alkaloid in young leaves (less than one year old) is **2**,^45^ and that **1** is only present in traces.^2^ When the plant in its second year reaches maturity, however, the content of **1** increases while **2** decreases.^45^ Our study shows that young leaves (6 months old) contained both **1** and **2**. Unfortunately, it was not possible to deduce which of these compounds was present in higher concentration. **2** is detectable without derivatization (Figure 3a), but when the tissue was treated with the derivatization agent, the signal intensity decreased (Figure 3b), an effect that was not observed in the fruits. In contrast, **1** was well detectable in the CA-treated tissue (Figure 3c). Even though the concentrations of **1** and **2** could not be assessed, the localization of these alkaloids could be determined. **2** was detected with a rather homogeneous distribution throughout the leaf surface. Interestingly, **6** was also detected on the leaf surface without derivatization (Figure 3a), with a slight intensification of the signal in the midrib. In contrast, **1** was mainly present in the base, midrib and vein regions as well as in the margin of the leaf (Figure 3c). In the derivatized tissue, **3** was present in margin, vein, and midrib (Figure 3c), in contrast to **4**, which was localized in the margin of the leaf (Figure 3b). This result may resolve the ambiguity previously presented between **3** and **4** localizations, since the distribution of these two signals was observed in different location of the tissue. However, there is still the possibility that the signal detected at *m*/*z* 142.1227 in the derivatized tissue belongs to **3** that was not completely derivatized. In the margin, we could also detect **5**, which after derivatization showed less delocalization (Figure 3b). l-Alanine was detectable throughout the leaf, with intense peaks at the tips but absent in the midrib (Figure 3c).

**Figure 4:**
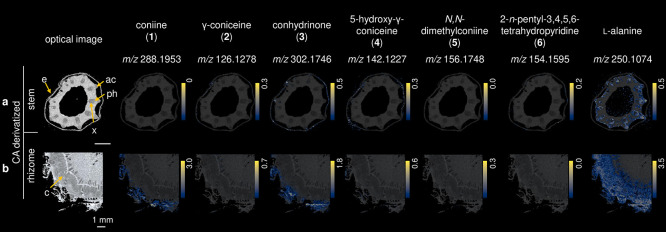
MALDI-MS imaging of different parts of *C. maculatum* after CA derivatization. (a) Stem and (b) rhizome. Overviews and descriptions of the tissue section of stem with angular collenchyma (ac), phloem (ph), epidermis (e), xylem (x), and rhizome cortex (c). Ion images of individual *m*/*z* values were generated on the same colorbar scale (yellow for the maximum percentage of ions and black for 0 ions detected) for visual comparison in terms of relative percentage of ion abundance.

The stem, rhizome, and root from a 6-months old *C. maculatum* plant were cut in transverse sections, derivatized with CA, and analyzed by MALDI-MSI (Figure 4). Remarkably, **1** and **3** have a similar distribution in the rhizome (Figure 4b). Surprisingly, despite the fact that **1** and **2** can readily be interconverted by an oxidoreductase, **2** was almost not detected in the rhizome, and **1** was not detected in stem. **3**/**4** and **6** were detected in stem, mainly in the epidermis of the tissue (Figure 4a). **2** and **5** were poorly detected in both tissues. In general, the distribution of the alkaloids was observed in the sections of tissues that are most exposed (for example, endocarp, margin, rhizome cortex or epidermis), which may support the use of these compounds as protection of the plant against insect attack.

In the stem, the biosynthetic precursor l-alanine was detectable mainly in the phloem (Figure 4a). Considering that this tissue is involved in long-distance amino acids transportation,^46^ the detection of l-alanine in the phloem is a confirmation of the correct localization analysis. Furthermore, l-alanine was detected in higher concentration in the cortex of the rhizome (Figure 4b), suggesting that l-alanine can be transported from the rhizome to the leaves through the phloem, which indeed is one of the possible ways of amino acid transportation in the plant.^47^ Additionally, l-alanine was detected in angular collenchyma of the stem tissue (Figure 4a).

In *C. maculatum* roots, it was not possible to detect any of the evaluated hemlock alkaloids or their CA derivatives (Figure S9). This is in agreement with literature reports that the hemlock alkaloids can only be detected in roots after the first year of growth.^45,48^

In conclusion, on-tissue derivatization permitted us to determine the localization of different alkaloids in *C. maculatum* via MALDI-MS imaging. This derivatization technique can be transferred to related small and/or volatile alkaloids in the tissues of other plants. The hemlock alkaloids show a distinct and differing localization, hinting at the presence or absence of specific biosynthesis enzymes in the individual parts of the tissues.

## Experimental Section

### Chemicals

Coniine was acquired from PhytoLab. CA and ClCiA were purchased from Sigma-Aldrich. TAHS was synthesized in two steps as described recently using *N*-succinimidyl carbonate (TCI chemicals), *N*,*N*-Dimethyl-*p*-phenylendiamine, and iodomethane (Sigma-Aldrich).^24^ HPLC-grade acetonitrile (MeCN) and methanol (MeOH) were purchased from Merck. Dry MeCN and CH_2_Cl_2_ were obtained from VWR. *N*,*N*-Diisopropylethylamine (DIPEA) was purchased from Carl Roth. Super-DHB was prepared by mixing 2,5-dihydroxybenzoic acid (DHB; obtained from TCI) and 2-hydroxy-5-methoxybenzoic acid (Sigma-Aldrich), in a weight ratio of 9:1. Gelatin was obtained from Sigma-Aldrich, carboxymethyl cellulose (CMC) from VWR, LC-MS grade formic acid (FA) from VWR, and trifluoroacetic acid (TFA) from Carl Roth.

### Plant Cultivation

*Conium maculatum* fruits were obtained from the botanical garden of the Martin-Luther-University Halle-Wittenberg. They were cultivated in sterile soil in a greenhouse, under a controlled temperature of 24 °C and a photoperiod of 16 h of light and 8 h of darkness. Six months old plants were harvested in October 2022. Germinated seeds were obtained on Murashige and Skoog medium including vitamins (Duchefa Biochemie bv), under dark conditions at 21 °C, and harvested on the ninth day after incubation before the cotyledons grew out of the fruit.

### Test Tube Reactions

Stock solutions of **1**, CA, and ClCiA were prepared in MeOH. For derivatization, 50 μL of CA (2 mg/mL, 11.2 mM) or 25 μL of ClCiA (2 mg/mL, 12.0 mM) was mixed with 50 μL of a solution of **1** (1 mg/mL, 7.9 mM). The mixtures were kept at 37 °C overnight. TAHS was dissolved in dry MeCN (1 mg/mL, 3.42 mM). For derivatization with TAHS, 15 μL of reagent dissolved was mixed with 4.4 μL of **1** (1 mg/mL, 7.9 mM) and 1.8 μL DIPEA (0.5% v/v in dry MeCN, pH 8–9). The mixture was kept at 55 °C overnight. All reactions were performed at 1000 *g* in a ThermoMixer MHR 23.

### LC-MS Analysis

Reaction solutions were analyzed by HRESIMS^2^ using a Q Exactive Plus Orbitrap Mass Spectrometer (Thermo Fisher Scientific), equipped with a heated electrospray ionization (ESI) interface, coupled to an UltiMate 3000 HPLC System (Thermo Fisher Scientific). Ionization was performed in positive and negative polarity modes. The capillary temperature was kept at 350 °C. The mass range was selected from *m*/*z* 50 to 750. Chromatography was performed on a Kinetex C18 column (50 × 2.1 mm, 2.6 μm, 100 Å; Phenomenex), eluted with H_2_O (A) and MeCN (B) (0.1% formic acid each), according to the following gradient program: 5% to 100% B (0–16 min), 100% B (16–20 min), flow rate 0.4 mL/min.

### MALDI Spot Analysis

One microliter of coniine (**1**) standard solution was applied to a 192-well ground steel MALDI target plate (MassTech) and coated with a solution of 2.5% v/v DIPEA in dry MeCN. The DIPEA solution was sprayed with 10 layers at 20 μL/min, RT, spray spacing of 2 mm, and spray velocity of 800 mm/min, to achieve a pH of 8–9 using a sprayer (SunCollect, SunChrome). Subsequently, the plate was sprayed with 26 mg/mL TAHS as derivatization agent in dry MeCN with 29 layers at 20 μL/min, RT, spray spacing of 2 mm, and spray velocity of 800 mm/min. The reaction was performed overnight at 55 °C in a hermetic chamber. After incubation, the plate was coated with super-DHB matrix at 25 mg/mL in dry MeCN and 2.5% v/v DIPEA, sprayed with 20 layers at 50 μL/min (the first three layers were sprayed with a reduced flow rate), RT, spray spacing of 2 mm, and spray velocity of 600 mm/min.

### MSI Sample Preparation

Stem, rhizome, and root from *C. maculatum* were harvested, embedded in a gelatin solution (10%, w/v), and immediately frozen in liquid nitrogen to form a solid block. Embedded samples were stored at −70 °C until sectioning. The stem, rhizome, and root tissues were sectioned with a thickness of 14 μm at −21 °C using a cryotome (MICROM HM 500 M, MICROM International GmbH) and thaw-mounted on VWR Superfrost Plus slides. Due to the complex texture and cellular structure of the dry fruits, these were embedded in gelatin/carboxymethyl cellulose (gelatin/CMC) (5%:2.5% w/v), preventing their flotation by using a viscous solution. The section thickness was 20 μm, and sections were cut with a transparent standard PP tape (Tesa) and placed on slides with a double-sided roller glue PS tape (Tesa), avoiding curling of the sections. The leaves were harvested and directly attached to the slide with double-side roller tape to avoid tissue rolling. The samples were dried in a desiccator with blue-indicating silica gel for 1 h, to avoid the evaporation and delocalization of the analytes. The samples were first observed using an inverse microscope (Axio Observer, Zeiss), and images were taken with an Axiocam 712 color digital camera for later comparison with the MSI results.

### On-Tissue Derivatization

On-tissue derivatization of **1** and related compounds was performed using a SunCollect sprayer. Fresh solutions of CA 8.0 mg/mL and ClCiA 7.5 mg/mL in HPLC-grade MeOH were sprayed as derivatization reagents on the tissues, with a solution of TAHS at 1 mg/mL in dry MeCN, and then the slides were incubated at 37 °C overnight. For TAHS derivatization, DIPEA and TAHS solutions were sprayed on the tissues, under the same conditions described above, and the slides were incubated overnight at 55 °C in a hermetic chamber. After incubation, CA- and ClCiA-derivatized tissue sections were coated with 25 mg/mL of super-DHB matrix in MeCN/H_2_O (1:1 v/v), and 0.1% TFA was sprayed with 20 layers at 50 μL/min, RT, spray spacing of 2 mm, and spray velocity of 600 mm/min. TAHS-derivatized tissue was coated with super-DHB matrix under the same conditions as described above. For all the slides, the first three layers were sprayed with a reduced flow rate of matrix solution.

### MALDI-MSI

Atmospheric pressure MALDI-MSI measurements were performed on a Fourier transform orbital trapping mass spectrometer (Q Exactive Plus, Thermo Fisher Scientific) equipped with an AP-MALDI (ng) UHR source (MassTech) with a laser spot size <10 μm. Imaging experiments were conducted in positive ion mode for 70–700 *m*/*z* with 140 000 resolution at *m*/*z* 200, one microscan, 5 × 10^6^ AGC target, 500 ms maximum injection time, 4.5 kV spray voltage, 450 °C capillary temperature, and 60% for the S-lens RF value. The MALDI source parameters were adjusted as follows: CSR mode (constant speed rastering), scanning velocity 2.3 mm/min for 20 μm and 3.45 mm/min for 30 μm pixel size, pulse rate 6 kHz, laser energy 31%. The centroid raw data were converted from the Thermo.raw files to.imzML using the MassTech imzML Converter (ng) 1.0.1 (merge strategy “Average”) and normalized by TIC. The converted files were analyzed with MSi Reader (v 1.01). All images were linear interpolated in order 3, with *m*/*z* ± 5 ppm tolerance. For SMART parameters,^49^ see Table S2. The ion images shown were selected as the most representative of at least two biological replicates per tissue.

### MS/MS Analysis of *C. maculatum* Fruit Extracts

Fruits of *C. maculatum* were dried and ground to a fine powder. Three milligrams of the powder was extracted with MeOH (5 mg of powder/mL). The fruit extract was centrifuged for 10 min at 10 000*g*. MS/MS analysis was performed by direct injection into a Thermo Q Exactive Plus mass spectrometer. The extract was analyzed by ESI at a flow of 10 μL/min, with a collision energy (CE) of 30 eV and MS^2^ isolation window of *m*/*z* 1.5 for extract solution.

## Supporting Information Available

Reaction schemes, HPLC-MS, MS/MS, MSI *C. maculatum* root, MSI *C. maculatum* fruit of **7** and **8**, theoretical and observed masses of derivatized metabolites (PDF)
