# Supplementary material for: Mass Spectrometry Imaging of Coniine and Other Hemlock Alkaloids after On-Tissue Derivatization Reveals Distinct Alkaloid Distributions in the Plant
Source: J Nat Prod. 2024 Jun 21;87(10):2376–83. doi: 10.1021/acs.jnatprod.4c00445 (PMC11519914; doi:10.1021/acs.jnatprod.4c00445)
Supplement: Supplementary file 1 — np4c00445_si_001.pdf [file np4c00445_si_001.pdf]

Supporting Information

Mass spectrometry imaging of coniine and other hemlock alkaloids after on-tissue derivatization reveals distinct alkaloid distributions in the plant

Diana A. Barrera-Adame[†,‡], *Sabine Schuster*[†,#], *Timo H. J. Niedermeyer*[‡,*]

[†]Department of Pharmaceutical Biology/Pharmacognosy, Institute of Pharmacy, Martin Luther University Halle-Wittenberg, 06120 Halle (Saale), Germany

[‡]Department of Pharmaceutical Biology, Institute of Pharmacy, Freie Universität Berlin, 14195 Berlin, Germany

[#]current affiliation: Simris Biologics GmbH, 12489 Berlin, Germany

*corresponding author, timo.niedermeyer@fu-berlin.de; +49 30 83875060

Scheme S1. Reaction of coniine with derivatization agents.. 3

Figure S1. Reaction of coniine with coniferyl aldehyde (CA) 4

Figure S2. Reaction of coniine with 4-chlorocinnamaldehyde (ClCiA).. 5

Figure S3. Reaction of coniine with *p*-*N*,*N*,*N*-trimethylammonioanilyl-*N'*-hydroxysuccinimidyl carbamate iodide (TAHS).. 6

Figure S4. MALDI-MS spectra (spot analysis) of the reaction of coniine with TAHS 7

Figure S5. Localization of coniine in *C. maculatum* fruit (underivatized / tissue treated with CA) 8

Figure S6. Localization of *N*,*N*-dimethylconiine and conmaculatin in *C. maculatum* fruit (tissue treated with CA)... 8

Figure S7. MSI visualization of the compound detected at *m/z* 140.1435 and *N*-methyl-pseudoconhydrine in *C. maculatum* fruit tissues (underivatized and derivatized) 9

Figure S8. Direct infusion mass spectrometry analysis of potentially novel hemlock alkaloid in *C. maculatum* fruit extract. 10

Figure S9. Mass spectrometry imaging of *C. maculatum* root tissue. 10

Table S1. Accurate mass measurement of derivatized alkaloids in *C. maculatum* tissue using MALDI-MSI. 11

Table S2. SMART parameters for the MS-Imaging analysis of *C. maculatum* tissues. 12

a

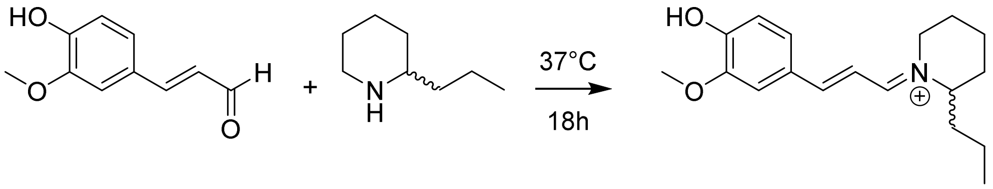

coniferyl aldehyde (CA) | (±) coniine (**1**) | (±) coniine-CA derivative

b

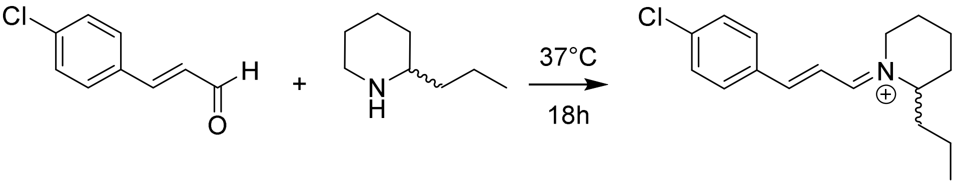

4-chlorocinnamaldehyde (ClCiA) | (±) coniine (**1**) | (±) coniine-ClCiA derivative

c

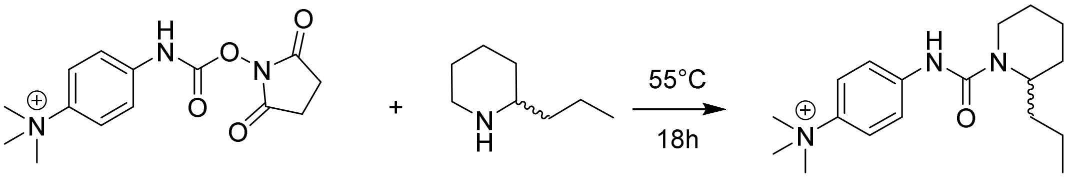

p-*N*,*N*,*N*-trimethylammonioanilyl- *N*'-hydroxysuccinimidyl carbamate iodide (TAHS) | (±) coniine (**1**) | (±) coniine-TAHS derivative

Scheme S1. Reaction of coniine (**1**) with derivatization agents: (a) CA, (b) ClCiA, and (c) TAHS.

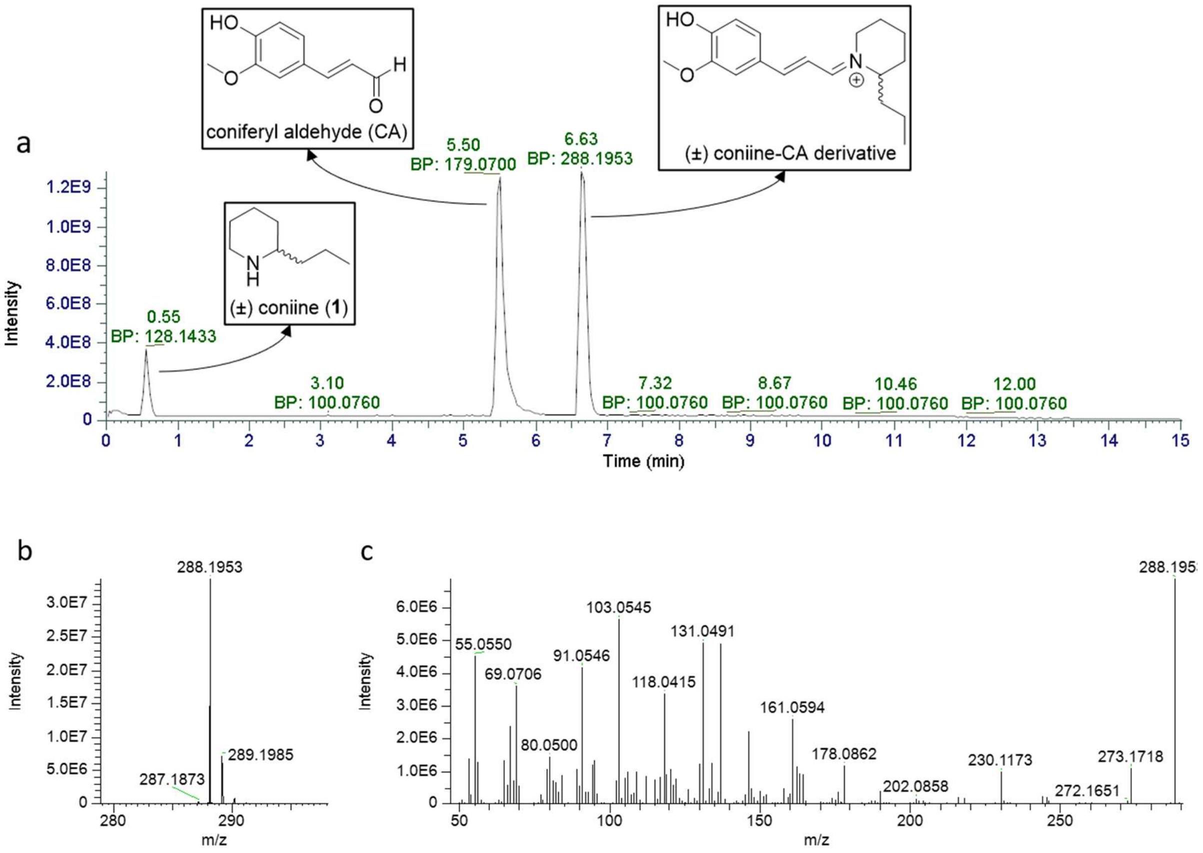

Figure S1. Reaction of coniine (**1**) with coniferyl aldehyde (CA). (a) LC-MS chromatogram, (b) MS spectrum of the reaction product, (c) MS/MS spectrum of the coniine-CA reaction product. All data generated in positive mode.

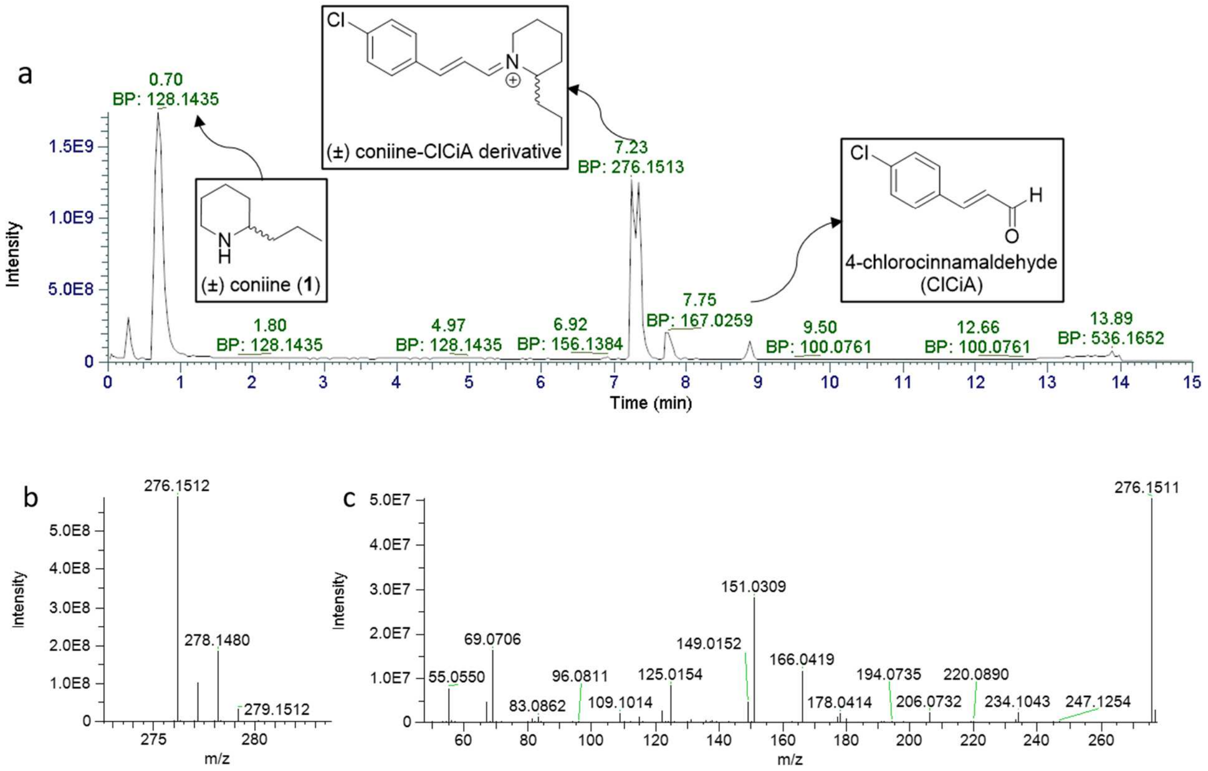

Figure S2. Reaction of coniine (**1**) with 4-chlorocinnamaldehyde (ClCiA). (a) LC-MS chromatogram, (b) MS spectrum of the reaction product, (c) MS/MS spectrum of the coniine-ClCiA reaction product. All data generated in positive mode.

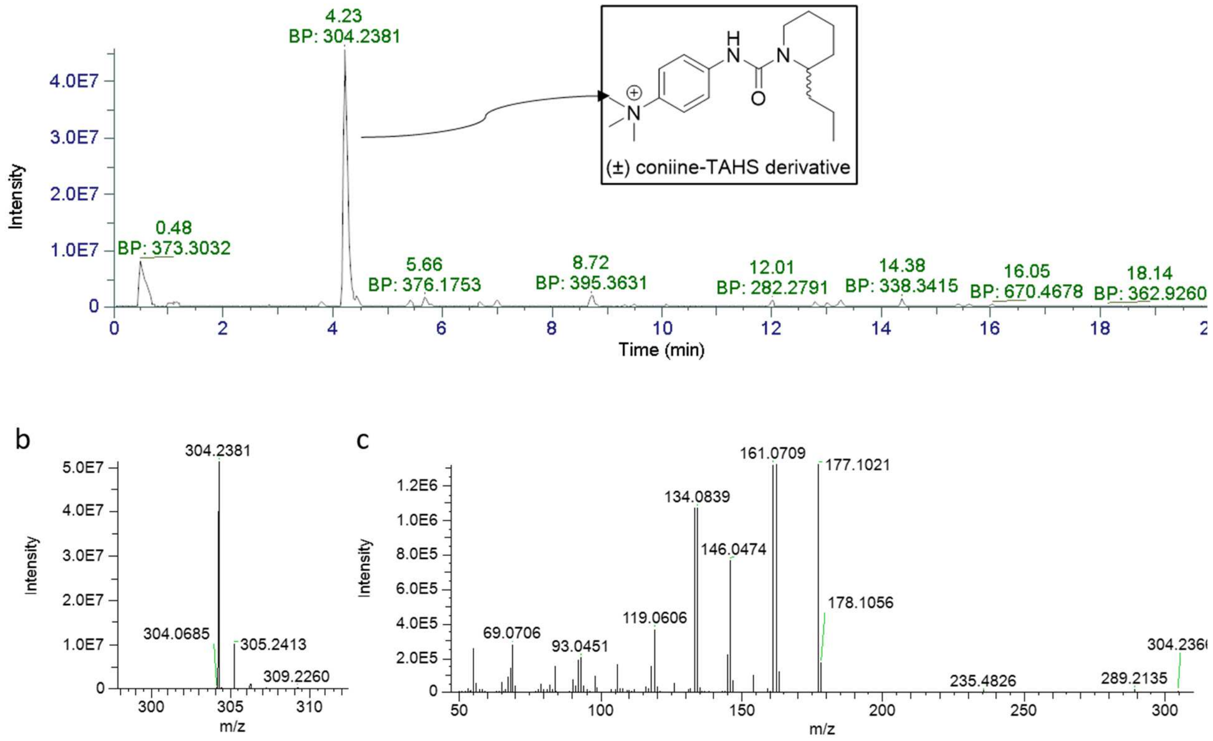

Figure S3. Reaction of coniine (**1**) with *p*-*N*,*N*,*N*-trimethylammonioanilyl-*N′*-hydroxysuccinimidyl carbamate iodide (TAHS). (a) LC-MS chromatogram, (b) MS spectrum of the reaction product, (c) MS/MS spectrum of the coniine-TAHS reaction product. All data were generated in positive mode.

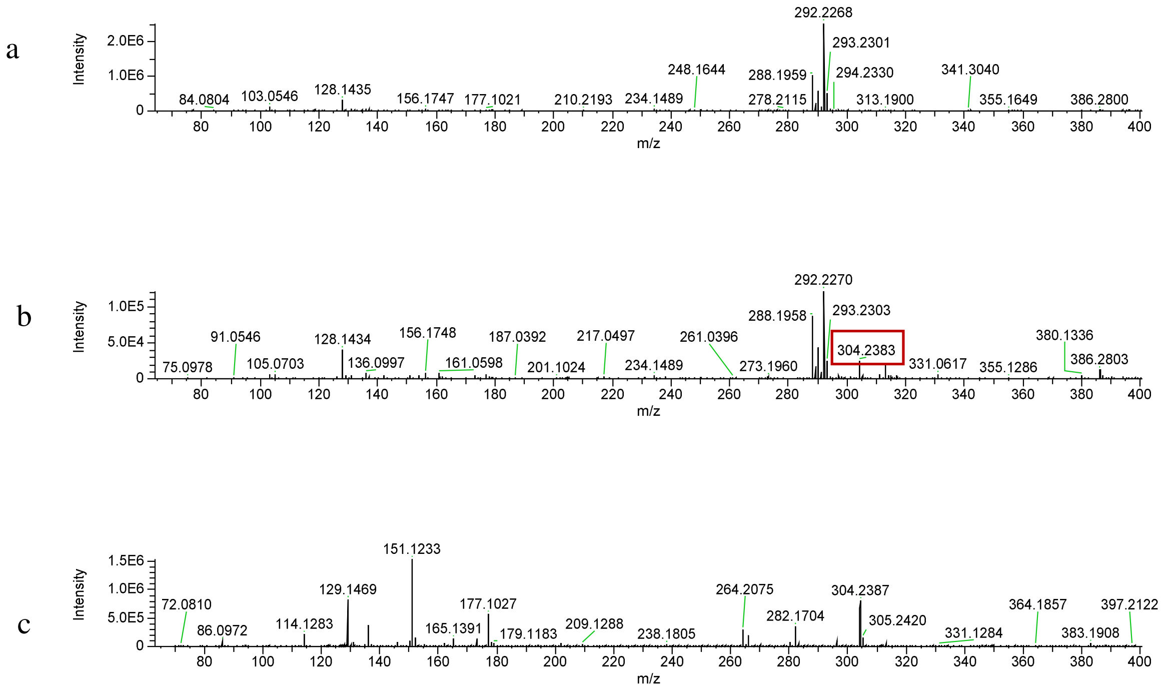

Figure S4. MALDI-MS spectra (spot analysis) of the reaction of coniine (**1**) with TAHS (a) in a test tube without DIPEA, (b) in a test tube with DIPEA (pH: 8-9), (c) on-slide with DIPEA (pH: 8-9)

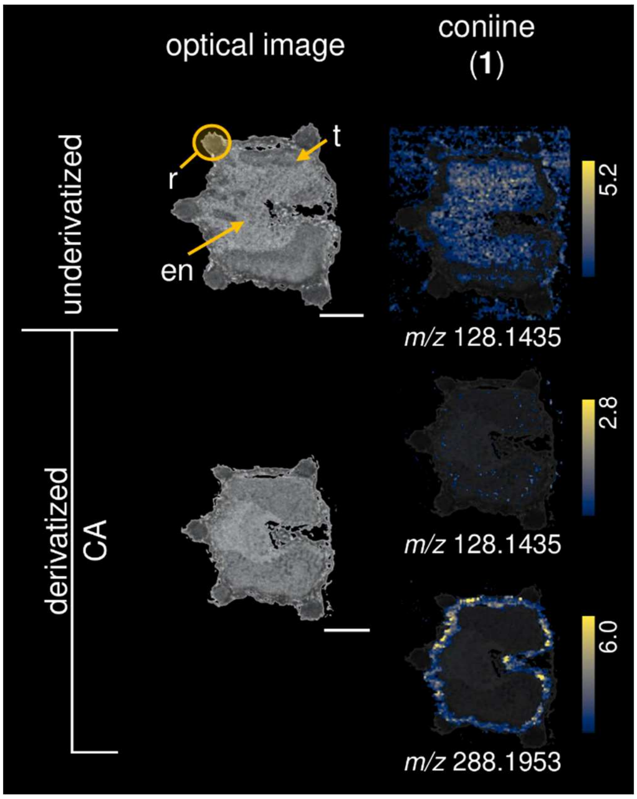

Figure S5. Localization of coniine (**1**) in *C. maculatum* fruit (underivatized / tissue treated with CA). Residual coniine is detectable after derivatization, showing that derivatization is not quantitative. Ion images of individual *m/z* values were generated on the same colorbar scale (yellow for the maximum percentage of ions and black for 0 ions detected) for visual comparison in terms of relative percentage of ion abundance.

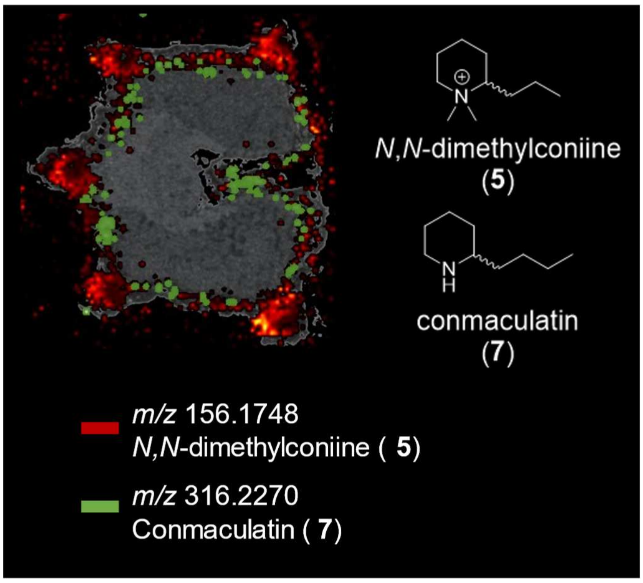

Figure S6. Localization of *N*,*N*-dimethylconiine (**5**) and derivatized conmaculatin (**7**) in *C. maculatum* fruit (tissue treated with CA).

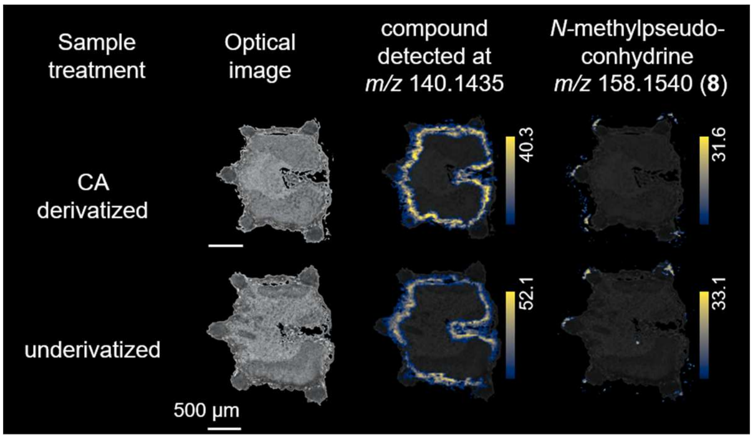

Figure S7. MSI visualization of the compound detected at *m/z* 140.1435 and *N*-methyl-pseudoconhydrine (**8**) in *C. maculatum* fruit tissues (underivatized and derivatized). Ion images of individual *m/z* values were generated on the same colorbar scale (yellow for the maximum percentage of ions and black for 0 ions detected) for visual comparison in terms of relative percentage of ion abundance.

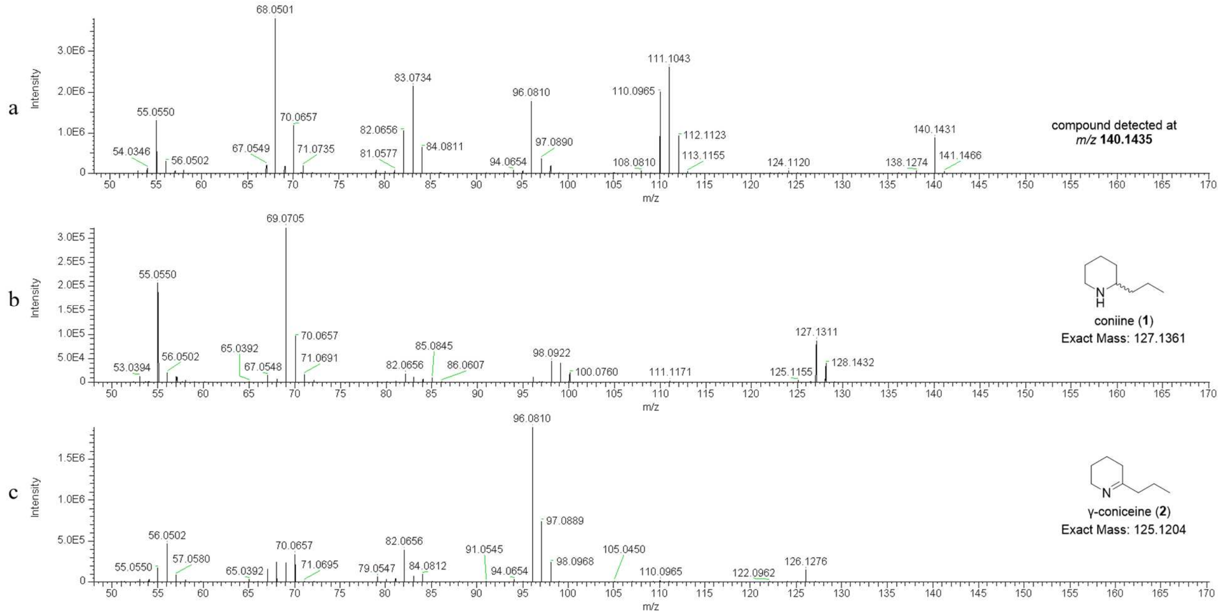

Figure S8. Direct infusion mass spectrometry analysis of potentially novel hemlock alkaloid in *C. maculatum* fruit extract. (a) MS/MS spectrum of the compound detected at *m/z* 140.1435, (b) MS/MS spectrum of coniine (**1**), (c) MS/MS spectrum of γ-coniceine (**2**). All data were generated in positive mode.

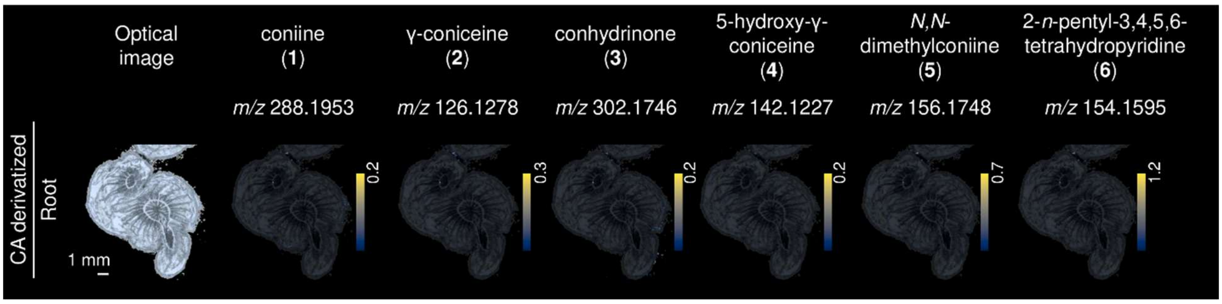

Figure S9. Mass spectrometry imaging of *C. maculatum* root tissue. Ion images of individual *m/z* values were generated on the same colorbar scale (yellow for the maximum percentage of ions and black for 0 ions detected) for visual comparison in terms of relative percentage of ion abundance.

Table S1. Accurate mass measurement of derivatized alkaloids in *C. maculatum* tissue using MALDI-MSI. *isobaric compounds. nd: not detected.

**Compound**	**Underivatized** $[M + H]^+$ ($[M]^+$ **for 5**)			**CA derivative** $[M + H]^+$			**ClCiA derivative** $[M + H]^+$			**TAHS derivative** $[M + H]^+$		
	theoretical	observed	error (ppm)	theoretical	observed	error (ppm)	theoretical	observed	error (ppm)	theoretical	observed	error (ppm)
coniine (**1**)	128.1434	128.1435	0.8	288.1958	288.1953	-1.7	276.1514	276.1513	-0.4	304.2383	304.2381	-0.7
γ-coniceine (**2**)	126.1277	126.1278	0.8	no reaction								
conhydrinone (**3**)	142.1226*	142.1227	0.7	302.1751	302.1746	-1.6	290.1306	290.1301	-1.7	318.2176	318.2178	0.6
5-hydroxy-γ-coniceine (**4**)	142.1226*	142.1227	0.7	no reaction								
N,*N*-dimethylconiine (**5**)	156.1747	156.1747	0.0	no reaction								
2-*n*-pentyl-3,4,5,6-tetrahydropyridine (**6**)	154.1590	154.1595	3.2	no reaction								
conmaculatin (**7**)	156.1747	156.1748	0.6	316.2277	316.2270	-2.2	not analyzed					
N-methylpseudoconhydrine or *N*-methylconhydrine (**8**)	158.1539	158.1540	0.6									
compound detected at *m/z* 140.1435	140.1434	140.1435	0.7	no reaction								
L-alanine	90.0550	n.d	-	250.1074	250.1074	0.0	238.0629	238.0630	0.4	266.1499	266.1498	-0.4

Table S2. SMART parameters for the MS-Imaging analysis of *C. maculatum* tissues.

(**S**): Spot size of the MALDI source: <10 µm. Experiments conducted in Constant Speed Rastering mode, scanning velocity 2.3 mm/min for 20 µm, 3.45 mm/min for 30 µm pixel size. Total scans see table below. (**M**): Molecular identification is based on high accuracy masses as described in Table S1, as well as selected MS/MS experiments done by direct infusion experiments as presented in Figure S8 and literature data on known alkaloids from *C. maculatum*. (**A**): The 10 compounds listed in Table S1 have been studied. (**R**): Resolution set to 140,000 at *m/z* 200. (**T**): Acquisition time see table below.

***C. maculatum* sample**	**Treatment**	**SMART parameters**	
fruits	underivatized	Step size	20 µm x 20 µm
		Total scans	18750
		Adquisition time (h)	2.9
	CA	Step size	20 µm x 20 µm
		Total scans	24000
		Adquisition time (h)	3.7
	CA (germinated)	Step size	20 µm x 20 µm
		Total scans	22950
		Adquisition time (h)	3.5
	ClCiA	Step size	20 µm x 20 µm
		Total scans	18750
		Adquisition time (h)	2.9
	TAHS	Step size	20 µm x 20 µm
		Total scans	2100
		Adquisition time (h)	3.2
leaves	underivatized	Step size	30 µm x 30 µm
		Total scans	86600
		Adquisition time (h)	13.1
	CA	Step size	30 µm x 30 µm
		Total scans	86600
		Adquisition time (h)	13.1
stem	CA	Step size	20 µm x 20 µm
		Total scans	66250
		Adquisition time (h)	10
rhizome	CA	Step size	20 µm x 20 µm
		Total scans	133225
		Adquisition time (h)	20
root	CA	Step size	40 µm x 40 µm
		Total scans	81250
		Adquisition time (h)	12.5
